# Repeat propofol anesthesia does not exacerbate plaque deposition or synapse loss in APP/PS1 Alzheimer’s disease mice

**DOI:** 10.1186/s12871-018-0509-5

**Published:** 2018-04-25

**Authors:** Adele Woodhouse, Carmen Maria Fernandez-Martos, Rachel Alice Kathryn Atkinson, Kelsey Anne Hanson, Jessica Marie Collins, Aidan Ryan O’Mara, Nico Terblanche, Marcus Welby Skinner, James Clement Vickers, Anna Elizabeth King

**Affiliations:** 1 Wicking Dementia Research and Education Centre , University of Tasmania, Hobart, Australia; 2 Tasmanian Health Service, Royal Hobart Hospital, Hobart, Australia; 3 Menzies Institute for Medical Research, University of Tasmania, Hobart, Australia; 4 Department of Health and Human Services Tasmania, Royal Hobart Hospital, Hobart, Australia; 5 School of Medicine, University of Tasmania, Hobart, Australia

**Keywords:** Alzheimer’s disease, β-amyloid plaques, Synapse, Synaptophysin, Glutamic acid decarboxylase

## Abstract

**Background:**

There is increasing interest in whether anesthetic agents affect the risk or progression of Alzheimer’s disease (AD). To mitigate many of the methodological issues encountered in human retrospective cohort studies we have used a transgenic model of AD to investigate the effect of propofol on AD pathology.

**Methods:**

Six month-old amyloid precursor protein/presenilin 1 (APP/PS1) transgenic AD mice and control mice were exposed to 3 doses of propofol (200 mg/kg) or vehicle, delivered at monthly intervals.

**Results:**

There was no difference in the extent of β-amyloid (Aβ) immunolabeled plaque deposition in APP/PS1 mice in vehicle versus propofol treatment groups. We also detected no difference in plaque-associated synapse loss in APP/PS1 mice following repeat propofol exposure relative to vehicle. Western blotting indicated that there was no difference in post-synaptic density protein 95, synaptophysin or glutamic acid decarboxylase 65/67 expression in control or APP/PS1 mice subjected to repeat propofol treatment relative to vehicle.

**Conclusions:**

These data suggest that repeat propofol anesthesia may not exacerbate plaque deposition or associated synapse loss in AD. Interestingly, this data also provides some of the first evidence suggesting that repeat propofol exposure in adult wild-type mice does not result in robust long-term alterations in the levels of key excitatory and inhibitory synaptic markers.

**Electronic supplementary material:**

The online version of this article (10.1186/s12871-018-0509-5) contains supplementary material, which is available to authorized users.

## Background

Alzheimer’s disease (AD) is the most common form of dementia and is forecast to become an increasing global burden with aging of the global populations [1]. AD is characterized by several pathological ‘hallmarks’ including β-amyloid (Aβ) plaques, neurofibrillary tangles, plaque-associated dystrophic neurites and neuropil threads. The majority of AD cases are sporadic in nature [2] and are likely caused by a combination of genetic susceptibility and environmental factors that interact to precipitate disease onset. Exposure to anesthetics is one such environmental factor that may contribute to the development and/or progression of AD. There is increasing interest in the link between anesthetic exposure, post-operative cognitive dysfunction (POCD) and the onset and progression of AD [3–15]. Notably, best practice for the use of anesthetics in people with mild cognitive impairment (MCI) and AD is not yet defined [3, 16].

As life expectancy is increasing, there is a rise in the number of elderly people undergoing anesthesia [10], however, data regarding the effects of anesthesia on the onset and progression of AD are contentious. Retrospective studies have reported that previous exposure to anesthesia was significantly correlated with an increased risk of AD in people over 80 years of age [17] and that there was an inverse correlation between anesthetic exposure before 50 years of age and the age of onset of AD [18]. However, other retrospective and meta-analyses studies have shown no association between anesthetic exposure and AD [14, 19, 20]. Moreover, there are substantial methodological issues to consider when interpreting the data from prospective randomized clinical trials; variations in perioperative/anesthetic procedures, impact of underlying conditions, lack of long-term follow up, poor controls, inadequate cognitive testing and surgery-associated inflammation [5, 10, 21].

POCD is a well-documented phenomena that shares mechanistic links with AD. POCD is common following general anesthesia in the elderly [7, 22, 23] and presents as memory loss, delirium, depression and impaired higher-level cognitive dysfunction [10]. POCD usually lasts only a few days, but POCD can persist for weeks and has been implicated in the development or progression of AD due to shared molecular mechanisms (increased CSF/brain Aβ levels and tau phosphorylation) [10, 22, 23]. Although the extent of POCD following particular anesthetic agents and surgery types varies [24]; aging [25, 26], pre-existing cognitive impairment [26, 27] and harboring the ε4 apolipoprotein allele [26, 28–30] all appear to play a role in the overall risk.

Propofol is a general anesthetic that is used for outpatient procedures (colonoscopy, endoscopy) through to extensive cardiac, hip and spinal surgeries. As older people often have several co-morbidities and/or chronic illness, they are commonly subjected to multiple surgical interventions. Propofol anesthesia has been reported to result in an increase [31–33], decrease [34, 35] and no change [36] in the incidence of POCD and dementia in humans. Likewise rodent studies have reported that exposure to propofol resulted in no change/decreased levels of Aβ [11, 37], and no change in plaque or tau pathology [38]. While behavioral studies following propofol anesthesia in rodents have observed no change [11, 38], decreased [39] or improved [13] cognitive function. However, when elderly patients were studied propofol use associated with POCD in approximately 50% of cases, even following minor surgery [40, 41].

Propofol acts as a GABA_A_ receptor agonist and a voltage-gated sodium channel antagonist [42–44] and it alters synapses in an age-dependent manner. In postnatal day 15 (P15) mice propofol exposure increased dendritic spine density in pyramidal neurons in the hippocampus (involved in memory formation and spatial navigation), the prefrontal cortex (involved in executive function, attention and memory) and the somatosensory cortex (which receives and processes sensory information from the body) [45, 46]. In contrast, propofol exposure at P5 reduced dendritic spine density in the prefrontal cortex and this was shown to be long lasting (up to P90) [45]. No studies to date have examined the impact of propofol anesthesia on synaptic structures in adult or aged subjects, which is particularly relevant due to the synaptic dysfunction and progressive plaque-associated synaptic loss that occurs in AD [47–49].

We investigated the impact of repeated propofol exposure on plaque deposition and synapses, in the APP/PS1 transgenic AD mouse that develop Aβ plaques and synaptic degeneration with aging. By using an AD mouse model we are able to mitigate many of the methodological issues encountered in retrospective cohort studies including variations in perioperative and anesthetic procedures, impact of underlying conditions and surgery-associated inflammation.

## Methods

### Mice

All experiments were approved by the Animal Ethics Committee of the University of Tasmania and performed according to the Australian Code of Practice for the Care and Use of Animals for Scientific Purposes (ethics number A12324). All experiments used male APP_SWE_/PSENdE9 (APP/PS1; [50], APP/PS1xYFPH [51]); Jackson Laboratory, USA, Strain B6.Cg-Tg(Thy1-YFP^+/−^) HJrs/J, Stock No. 003782), YFPH and C57Bl/6 wild-type littermate mice at 6 months of age. APP/PS1 and APP/PS1xYFPH mice over-express APP harboring familial AD mutations that result in increased expression of the APP cleavage product Aβ, and the development of Aβ plaques and synaptic degeneration with aging [47, 50, 52]. Amyloid plaques and memory deficits appear at 6–7 months of age in APP/PS1 mice and are abundant by 9 months, however, APP/PS1 mice do not develop the neurofibrillary pathology characteristic of human AD [50]. These mice represent a dynamic model of Aβ plaque and synaptic pathology that can be experimentally increased [53, 54] or decreased [55]. All animals were housed in standard conditions (12 h light/dark cycle, 20 °C) with ad libitum access to food and water.

### Anesthetic exposure

Six-month-old APP/PS1, APP/PS1xYFPH, YFPH and wild-type mice were randomly allocated to propofol or vehicle treatment groups; and administered propofol (200 mg/kg; Norbrook® Laboratories, Australia PTY Ltd) diluted in Intralipid® (Fresenius Kabi Ltd., UKm 0338–0519) or a vehicle control (Intralipid®, Fresenius Kabi Ltd., UKm 0338–0519) intraperitoneally (IP) three times at 6 (representing pathology onset time-point), 7 and 8 months of age. All animals were then terminally anesthetized and perfused at 9 months of age. The dose of propofol administered (200 mg/kg) results in the loss of the righting reflex in > 95% of adult mice [56]. During anesthetic exposure the mice breathed spontaneously and were kept warm on a heating pad until they recovered and were mobile. Information regarding anesthetic induction and emergence time (indicating by the presence of the righting reflex) were collected for each propofol exposure. Statistical analysis of the average emergence time was performed; a student’s t-test (2 tailed, type 3; Microsoft Excel) with a *p* value of 0.05 considered statistically significant. Data generated from APP/PS1 or wild-type mice were not significantly different from that of APP/PS1xYFPH or YFPH mice, respectively; thus these data were pooled and designated APP/PS1 and control experimental groups.

### Immunohistochemistry and analysis

Mice were terminally anesthetized (sodium pentobarbitone, 110 mg/kg, IP) and transcardially perfused (4% paraformaldehyde in 0.01 M phosphate buffered saline (PBS)). Brains were then cryoprotected (18% and 30% sucrose) and 40 μm serial coronal sections were cut on a cryostat (Leica CM 1850). Immunohistochemistry for Aβ plaques (MOAβ-2 antibody: 1:2000, Novus Biologicals, cat no. NBP2–13075) was performed as previously described [57]. Propofol and vehicle treated APP/PS1 positive mice (*n* = 8 and 6) and APP/PS1 negative mice (n = 8 and 7) were used for plaque analysis. Immunolabeling for synaptic puncta (synaptophysin antibody: 1:200, Millipore, cat no. AB9272, NIF Antibody Registry AB_570874) and staining with thioflavin-S (Sigma-Aldrich, cat no. T-1892) were performed as previously described [53, 57]. Propofol and vehicle treated APP/PS1 mice (*n* = 3 for both) were used for synaptic puncta analysis. Negative control experiments (omitting primary antibodies) eliminated all immunoreactivity. Primary antibodies were visualized with AlexaFluor goat anti-mouse/rabbit secondary antibodies (1:500, Molecular Probes).

Images of MOAβ2-labeling were captured on a Leica DM LB2 microscope (NIS-Elements D Imaging Software, Nikon Instruments) as previously described [57]. Images of synaptophysin immunolabeling and thioflavin-S staining were captured on a Perkin-Elmer Ultraview VOX spinning disk confocal imaging system (Volocity 6.3 software, Perkin-Elmer) with the same laser power and exposure settings, as previously described [53]. All image collection and subsequent image analysis was performed by an investigator blinded to treatment group allocation. Analysis of Aβ plaques and thioflavin-S plaque-associated synapse loss were conducted with a custom unbiased image segmentation plugin for ImageJ based on a random-forests machine learning algorithm to segment images as plaques and synaptic puncta or background pixels [58]. The classifier was trained using a random selection of cropped images from the data set that were annotated with examples of plaques/synaptic puncta and background pixels to produce a forest of 50 trees with a maximum depth of 9 nodes. Each tree considered only a random bag of 5% of the training pixels, sampled with replacement. All analysis was conducted blinded to animal genotype/treatment group. Statistical analysis of the plaque density, average plaque size, plaque load, the density of and percentage area occupied by synaptophysin-immunoreactive synaptic puncta was performed using the student’s t-test (2 tailed, type 3; Microsoft Excel) for analysis, with a *p* value of 0.05 considered statistically significant. The *n* required to power this study could not be predicted a priori as no previous study had investigated repeat propofol exposure in the APP/PS1 mouse model at 6 months of age with the same dosing regime as the current study, nor had any previous studies used the custom unbiased image segmentation plugin for ImageJ (based on a random-forests machine learning algorithm) to segment images as plaques and synaptic puncta or background pixels [58]. Therefore, for the maximum value of change detectable for each of our datasets refer to Additional file 1. Data are presented as the mean ± SEM. Figures were prepared in Adobe Photoshop and Adobe Illustrator, with brightness and contrast being enhanced for clarity consistently across images.

### Western blot

Propofol and vehicle treated APP/PS1 (*n* = 3 and 2) and wild-type control mice (n = 3 for both) were used for Western blot analysis. At 9 months of age, mice were terminally anesthetized (sodium pentobarbitone, 110 mg/kg IP) and transcardially perfused (0.01 M PBS), the neocortex was then quickly removed and frozen in liquid nitrogen. Samples were homogenized in RIPA buffer (Sigma-Aldrich) containing a proteinase inhibitor cocktail (Roche) as previously [59]. Denatured proteins samples (20 μg) were electrophoresed into Bolt® Bis-Tris Plus gels (Invitrogen), transferred to PVDF membranes (BioRad), and incubated overnight in primary antibody solution at 4 °C. Primary antibodies used for Western blot were mouse anti-PSD-95 (1:1000; Abcam cat no. ab2723, NIF Antibody Registry AB_303248), rabbit anti-synaptophysin (1:1000; Millipore cat no. AB9272, NIF Antibody Registry AB_570874), mouse anti-GAD65 (1:1000; Abcam cat no. ab26113, NIF Antibody Registry AB_448989) and mouse anti-GAD67 (1:1000; Millipore cat no. MAB5406, NIF Antibody Registry AB_2278725). PSD-95 is a marker of the postsynaptic density of excitatory synapses, synaptophysin is a pre-synaptic marker of excitatory and inhibitory synapses, while GAD65 and GAD67 are markers of presynaptic inhibitory synapses. Membranes were washed and incubated with the corresponding anti-rabbit or anti-mouse horseradish peroxidase (HRP)-conjugated secondary antibody (1:7000; Dako) for 2 h at room temperature. Immunoreactive bands were visualized with enhanced chemiluminescence (ECL) solution using Luminata Forte Western horseradish peroxidase (HRP) substrate (Merck Millipore, Billerica, MA, USA). Membranes were then stripped and re-probed with a mouse anti-GAPDH (1:1000, Millipore cat no. MAB374, NIF Antibody Registry AB_2107445), as a loading control. Western blot images were captured with the same exposure for all experimental groups for each protein of interest.

## Results

### No difference in emergence time from propofol anesthesia in APP/PS1 and control mice

To determine whether there was any difference in the response to propofol anesthesia the emergence time, indicated by the return of the righting reflex, was assessed for propofol treated control and APP/PS1 mice. There was no significant difference in the average emergence time from propofol anesthesia between APP/PS1 (43.1 ± 8.9 min, *n* = 9) and control (64.0 ± 17.6 min, *n* = 7) mice (*p* > 0.05).

### Repeat propofol exposure did not alter plaque deposition in the cortex of APP/PS1 mice

**Fig. 1:**
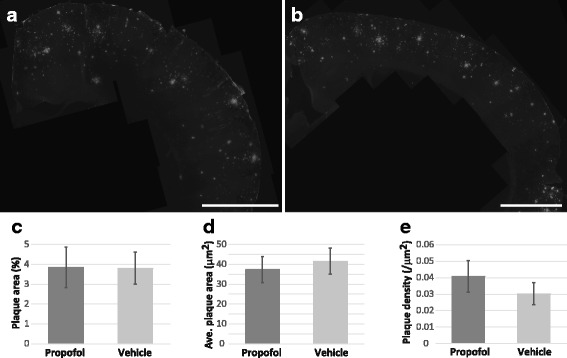
No difference in Aβ plaque load between propofol (*n* = 8) and vehicle (*n* = 6) treated APP/PS1 mice. **a** An example image of Aβ plaque immunoreactivity in the cortex of an APP/PS1 mouse following repeated propofol exposure. **b** A representative image of Aβ plaque immunolabeling the cortex of an APP/PS1 mouse following treatment with vehicle. **c** Bar graph showing the percentage of the cortex occupied by Aβ plaques in propofol and vehicle treated APP/PS1 mice. **d** Bar graph showing the average size (μm^2^) of Aβ plaques in the cortex of APP/PS1 in the propofol and vehicle treatment groups. **e** Bar graph showing the average density of Aβ plaques (/μm^2^) in the cortex of propofol and vehicle exposed APP/PS1 mice. All data is shown as mean ± SEM. Scale bar = 500 μm

We assessed the impact of repeat propofol anesthesia on Aβ plaque deposition in propofol and vehicle treated APP/PS1 mice. There was no significant difference in the Aβ plaque (MOAβ-2-labeled) load between vehicle (3.80 ± 0.8%) and propofol (3.85 ± 1.0%) treated APP/PS1 mice compared to control mice (p > 0.05, Fig. 1, Table 1). There was also no significant difference in the average size or density of Aβ plaques between vehicle (41.6 ± 6.5 μm^2^, 0.030 ± 0.007/μm^2^, respectively) or propofol (37.4 ± 6.6 μm^2^, 0.041 ± 0.009/μm^2^, respectively) treated APP/PS1 mice (*p* > 0.05, Fig. 1, Table 1). No Aβ plaques were observed in the vehicle or propofol treated control mice.

**Table 1:** Mean values with standard error (SEM) for Aβ plaque deposition and synaptophysin immunoreactivity data

	APP/PS1	APP/PS1
	Propofol	Vehicle
Aβ plaque load	3.85 ± 1.0%	3.80 ± 0.8%
Aβ plaque size	37.4 ± 6.6 μm^2^	41.6 ± 6.5 μm^2^
Aβ plaque density	0.041 ± 0.009/μm^2^	0.030 ± 0.007/μm^2^
Synapse density R1	0.366 ± 0.03/μm^2^	0.359 ± 0.017/μm^2^
Synapse % area R1	1.63 ± 0.17%	1.55 ± 0.12%,
Synapse density R2	0.234 ± 0.085/μm^2^	0.268 ± 0.060/μm^2^
Synapse % area R2	2.37 ± 0.90%	2.57 ± 0.63%

Aβ plaque load and Aβ plaque density were determined using the MOAβ-2 antibody in propofol (*n* = 8) and vehicle (*n* = 6) treated APP/PS1 mice

Synapse density and percentage area were analyzed in vehicle (*n* = 3) and propofol (*n* = 3) treated APP/PS1 mice in: Region 1 (R1), < 40 μm from Thioflavin-S plaques: and Region 2 (R2) = 40-80 μm away from Thioflavine-S plaques

### Repeat propofol exposure did not alter plaque-associated synaptic degeneration or the expression of synaptic proteins in APP/PS1 mice

**Fig. 2:**
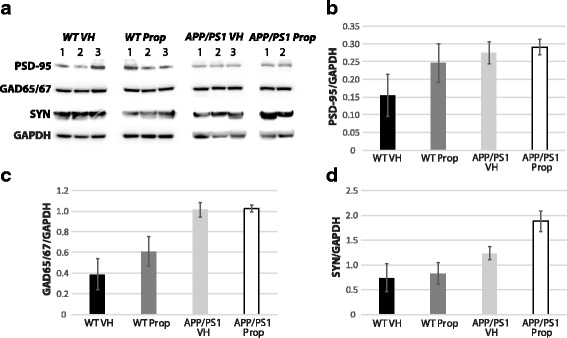
No change in synaptic proteins between propofol and vehicle treated APP/PS1 (*n* = 3 and 2) and control wild-type (*n* = 3 for both) mice. Western blot analysis (**a**) showed no robust change in the expression levels of PSD-95, GAD65/67 and synaptophysin (SYN) in wild-type or APP/PS1 mice treated multiple times with propofol (Prop) or vehicle (VH). Bar graphs show the mean intensity ± SEM of PSD-95 (**b**), GAD65/67 (**c**) and synaptophysin (**d**) normalized to GAPDH

As propofol increases GABAergic activity and decreases neuronal intrinsic excitability we assessed whether repeat propofol exposure altered the levels of excitatory and inhibitory synaptic markers in control and APP/PS1 mice as well as plaque-associated synaptic degeneration in APP/PS1 mice. Western blot analysis showed no robust difference in the expression levels of post-synaptic density protein 95 (PSD-95), synaptophysin and glutamic acid decarboxylase 65/67 (GAD65/67) in the cortex between vehicle and propofol treated control or APP/PS1 mice (Fig. 2). There was no difference in the density of, or percentage area occupied by, synaptophysin-immunoreactive synaptic puncta in the cortex < 40 μm (region 1) from thioflavin-S plaques in vehicle (0.359 ± 0.017/μm^2^, 1.55 ± 0.12%, respectively) or propofol (0.366 ± 0.03/μm^2^, 1.63 ± 0.17%, respectively) treated APP/PS1 mice (*p* > 0.05; Table 1). There was also no difference in the density of, or percentage area occupied by synaptophysin-immunoreactive synaptic puncta in the cortex between 40 and 80 μm (region 2) away from thioflavin-S plaques in vehicle (0.268 ± 0.060/μm^2^, 2.57 ± 0.63%, respectively) or propofol (0.234 ± 0.085/μm^2^, 2.37 ± 0.90%, respectively) treated APP/PS1 mice (*p* > 0.05; Table 1).

## Discussion

APP/PS1 and control mice were exposed to an average of 54 min of propofol anesthesia repeatedly at 6, 7 and 8 months of age to determine the effect of repeat propofol anesthesia on Aβ plaque pathology and synapses. We detected no difference in plaque load, plaque-associated synapse loss or the expression of excitatory and inhibitory synaptic markers in APP/PS1 mice repeatedly anesthetized with propofol compared to APP/PS1 vehicle controls. We also provide some of the first evidence suggesting that repeat propofol exposure in adult wild-type mice does not result in robust long-term alterations in the levels of PSD-95, synaptophysin and GAD65/67.

Repeat propofol exposure did not result in a difference in Aβ plaque load, plaque size or plaque density in APP/PS1 mice in the current study. These data are in contrast to the decreased Aβ load reported in 15-monthold Tg2576 AD mice and Aβ levels in 18-month-old wild-type mice following repeated propofol exposure [11, 37]. Differences in the AD mouse model used (Tg2576) [11], the age of the animals, the dose of propofol used (26 mg/kg bolus and 2 mg/kg/min infusion [11] 50 mg/kg bolus [37]), as well as the propofol dosing regime [11, 37] may account for the differing impact on Aβ dynamics reported between this and previous studies. Our study focused on the impact of repeat propofol anesthesia between 6 and 9 months of age as Aβ plaque deposition occurs at a rapid rate in APP/PS1 mice during this time [60]. It is possible that the impact of propofol exposure in older APP/PS1 mice may differ. Indeed, Shao and colleagues (2014) observed improved performance in the Morris Water Maze in 22-month-old APP/PS1 and aged wild-type mice following weekly propofol exposure for 3 months [13]. In keeping with our data, behavioral studies have observed that repeated propofol exposure resulted in no difference in Y maze performance in Tg2576 AD mice [11]. Similarly, rat studies have reported that one propofol exposure did not significantly alter olfactory learning in aged rats [61], while repeat propofol exposure improved inhibitory avoidance performance [62]. Interestingly, a recent human prospective study did not detect a difference in cerebrospinal fluid levels of Aβ1–42, total tau or phosphorylated tau between propofol exposed and control MCI groups [9]. Furthermore, at 2 year follow-up no difference in the rate of MCI progression or conversion to AD between propofol exposed (spinal surgery) MCI cases compared to non-surgery MCI controls was detected [9].

Notably, as APP/PS1 mice do not exhibit substantial tau pathology, it is possible that propofol may still influence the onset and/or progression of AD tau pathology. However, a recent study investigated the impact of ~ 30 min of propofol exposure in 3xTgAD mice, which develop both Aβ and tau pathology indicates that this is not the case [38]. Mardini and colleagues detected no difference in performance between propofol exposed and control 3xTgAD mice in the Morris Water Maze both 3 weeks and 16 weeks following propofol exposure [38]. Likewise, at 18 weeks following propofol exposure there was no change in Aβ plaque load, phosphorylated-tau aggregation or the number of activated microglia between the propofol exposed and control 3xTgAD mice [38]. This suggests that transient increases in tau hyperphosphorylation in wild-type and transgenic AD mice following a single propofol exposure [63, 64] does not result in long-lasting sequelae.

As propofol is a GABA_A_ agonist we investigated the impact of repeat propofol anesthesia on the synaptic degeneration and dysfunction that occurs in AD [47–49]. There was no exacerbation of the plaque-associated synaptic loss in APP/PS1 mice treated with propofol versus vehicle, suggesting that repeat propofol exposure does not exacerbate synaptic degeneration. Furthermore, we provide some of the first data that indicates that repeat propofol anesthesia in adult mice does not have a robust long term effect on the levels of key excitatory and inhibitory synaptic proteins; PSD-95, synaptophysin and GAD65/67 were not altered between propofol and vehicle treated cohorts of APP/PS1 or control mice. This is pertinent as recent animal studies have suggested that the dysfunction of inhibitory neuron networks contribute to aberrant excitatory neuronal activity in AD [48, 49], and decreased levels of GABA_A_ receptor subunits have been also observed in human AD [65–69]. These data in adult mice are also in contrast to the long-lasting reduction in spine density in the prefrontal cortex observed following propofol exposure at P5 [45], as well as the propofol-induced increase in dendritic spine density in pyramidal neurons in the hippocampus and somatosensory cortex observed at P15 [46]. However, it should be noted that the design of these developmental studies [45, 46] differed from the current study in several ways including; the propofol dosing regime (40-50 mg/kg propofol initial bolus with 1–1.5 hourly injections of 20-25 mg/kg for a single 5–6 h propofol exposure), analysis of synapses (spine density analysis versus synaptic puncta analysis) and the brain region analyzed (prefrontal cortex, somatosensory cortex and hippocampus versus cingulate, motor and somatosensory cortex); which may account for differences in the synaptic data.

## Conclusions

Our data, along with other studies investigating propofol exposure and AD, suggest that propofol is unlikely to exacerbate plaque deposition or synapse alterations in AD. However, as the APP/PS1 mouse model does not develop extensive tau pathology, it is important to note that propofol may still impact neural health and could mitigate the onset or progression of AD. This study also provides some of the first data to demonstrate that key synaptic markers are not altered in adult wild-type mice following repeat propofol exposure.

## Additional file

Additional file 1: The maximum change detectable for each dataset with 80% power (Graphpad Statmate 2). (DOCX 12 kb)

## Abbreviations

AD: Alzheimer’s disease
APP: Amyloid precursor protein
Aβ: β-amyloid
GABA: Gamma aminobutyric acid receptor A
GAD65: Glutamic acid decarboxylase isoform 65
GAD67: Glutamic acid decarboxylase isoform 67
GAPDH: Glyceraldehyde 3-phosphate dehydrogenase
IP: Intraperitoneally
MCI: Mild cognitive impairment
PBS: Phosphate buffered saline
POCD: Post-operative cognitive dysfunction
PS1: Presenilin 1
PSD-95: Post synaptic density protein 95
YFPH: Yellow fluorescent protein high
