# Supplementary material for: Repeat propofol anesthesia does not exacerbate plaque deposition or synapse loss in APP/PS1 Alzheimer’s disease mice
Source: BMC Anesthesiol. 2018 Apr 25;18:47. doi: 10.1186/s12871-018-0509-5 (PMC5921792; doi:10.1186/s12871-018-0509-5)
Supplement: Supplementary file 1 — The maximum change detectable for each dataset with 80% power (Graphpad Statmate 2). (DOCX 12 kb) [file 12871_2018_509_MOESM1_ESM.docx]

**Additional File 1**

**The maximum change detectable for each dataset with 80% power (Graphpad Statmate 2).**

| **Dataset** | **Change detected with 80% power** |
| --- | --- |
| Aβ plaque load | 4.21% |
| Aβ plaque size | 28.9 µm2 |
| Aβ plaque density | 0.038/µm2 |
| Synapse density R1 | 0.13/µm2 |
| Synapse % area R1 | 0.73% |
| Synapse density R2 | 0.37/µm2 |
| Synapse % area R2 | 3.91% |
